# Long noncoding RNA *HOTAIR* is relevant to cellular proliferation, invasiveness, and clinical relapse in small-cell lung cancer

**DOI:** 10.1002/cam4.220

**Published:** 2014-03-03

**Authors:** Hiroshi Ono, Noriko Motoi, Hiroko Nagano, Eisaku Miyauchi, Masaru Ushijima, Masaaki Matsuura, Sakae Okumura, Makoto Nishio, Tetsuro Hirose, Naohiko Inase, Yuichi Ishikawa

**Affiliations:** 1 Division of Pathology, The Cancer Institute, Japanese Foundation for Cancer ResearchTokyo, Japan; 2 Department of Integrated Pulmonology, Tokyo Medical and Dental UniversityTokyo, Japan; 3 Bioinformatics Group, Genome Center, Japanese Foundation for Cancer ResearchTokyo, Japan; 4 Division of Cancer Genomics, The Cancer Institute, Japanese Foundation for Cancer ResearchTokyo, Japan; 5 Thoracic Oncology Center, The Cancer Institute Hospital, Japanese Foundation for Cancer ResearchTokyo, Japan; 6 Institute for Genetic Medicine, Hokkaido UniversitySapporo, Japan

**Keywords:** *HOTAIR*, invasiveness, lincRNA, proliferation, small-cell lung cancer

## Abstract

Small-cell lung cancer (SCLC) is a subtype of lung cancer with poor prognosis. To identify accurate predictive biomarkers and effective therapeutic modalities, we focus on a long noncoding RNA, *Hox* transcript antisense intergenic RNA (*HOTAIR*), and investigated its expression, cellular functions, and clinical relevance in SCLC. In this study, *HOTAIR* expression was assessed in 35 surgical SCLC samples and 10 SCLC cell lines. The efficacy of knockdown of *HOTAIR* by siRNA transfection was evaluated in SBC-3 cells in vitro, and the gene expression was analyzed using microarray. *HOTAIR* was expressed highly in pure, rather than combined, SCLC (*P* = 0.012), that the subgroup with high expression had significantly more pure SCLC (*P* = 0.04), more lymphatic invasion (*P* = 0.03) and more relapse (*P* = 0.04) than the low-expression subgroup. The knockdown of *HOTAIR* in SBC-3 cells led to decreased proliferation activity and decreased invasiveness in vitro. Gene expression analysis indicated that depletion of *HOTAIR* resulted in upregulation of cell adhesion-related genes such as *ASTN1*, *PCDHA1*, and mucin production-related genes such as *MUC5AC*, and downregulation of genes involved in neuronal growth and signal transduction including *NTM* and *PTK2B*. Our results suggest that *HOTAIR* has an oncogenic role in SCLC and could be a prognostic biomarker and therapeutic target.

## Introduction

Lung cancer is a leading cause of cancer death worldwide 1. Small-cell lung cancer (SCLC) is an aggressive subtype, characterized by a neuroendocrine nature, which represents ∼15% of all newly diagnosed lung cancers 2. SCLC patients have a poor prognosis compared with non–small-cell lung cancers (NSCLCs) due to more rapid growth and more frequent recurrence. Although the survival of NSCLC patients has been significantly improved by targeted chemotherapy, there are currently no targeted drugs effective against SCLC. To identify accurate predictive biomarkers and to develop effective therapeutic modalities, elucidation of molecular mechanisms underlying the rapid growth and the high propensity for relapse of SCLC is essential.

Advances in experimental technology have been applied to studies of malignant tumors including SCLC 3. In particular, application of modern genetic profiling technology to the study of noncoding RNAs has revealed a crucial role for these molecules in tumor cell regulation. Similar to short regulatory noncoding RNAs (ncRNAs), such as microRNAs, many long intergenic ncRNAs (lincRNAs) have been found to be important by functioning as the interface between DNA and specific chromatin remodeling activities 3–7. These lincRNAs are involved in diverse cellular processes, including cell-cycle regulation, immune surveillance, and stem cell pluripotency.

*Hox* transcript antisense intergenic RNA (*HOTAIR*) is one of the few biologically well-studied lincRNAs 8,9. Previous studies have demonstrated that *HOTAIR* is transcribed from *HoxC* gene as an antisense transcript, and binds polycomb repressive complex 2 (PRC2) and LSD1-CoREST-REST complex as scaffolds, leading to catalyzing trimethylation of H3K27 and spontaneous demethylation of H3K4, and to repressing transcription of *HoxD* genes 9. REST (RE1 silencing transcriptional factor, also called neuron-restrictive silencer factor) and its corepressors negatively regulate neurogenesis and contribute to the maintenance of pluripotency of neural cells 10, whereas LSD1 (lysin-specific demethylase 1) regulates neural stem cell proliferation 11. In relation to DNA methylation, EZH2, a compartment of PRC2, directly interacts with DNA methyltransferases (DNMT1, DNMT3A and DNMT3B). This interaction is necessary for maintenance of DNA methylation and stable repression of specific genes, including many tumor suppressors 12. In fact, 20% of the lincRNAs have been shown to associate with PRC2. The homeobox-containing genes as targets of *HOTAIR* are a family of transcriptional regulators encoding DNA-binding homeodomains involved in the control of normal development 4,5. Also, aberrant expression of homeobox genes is associated with both morphological abnormalities and carcinogenesis 6,7. Moreover, a most recent study suggested that the role of *HOTAIR* in tumorigenesis occurs through triggering epithelial-to-mesenchymal transition (EMT) and acquiring stemness and its maintenance 13.

Although *HOTAIR* and its association in cancer metastasis and prognosis of diverse cancers have been suggested in several studies 14–22, its functions in SCLC remain unclear. In this study, we investigated the role of *HOTAIR* for cellular proliferation and patients' prognosis to develop a biomarker and a new target for therapy of SCLC.

## Materials and Methods

### Clinical samples and cell lines

Between January 1995 and December 2010, 3460 patients with primary lung cancer underwent surgery at the Cancer Institute Hospital of Japanese Foundation for Cancer Research (JFCR), Tokyo, Japan. Since SCLC is usually inoperable, only 55 (1.6%) cases had been diagnosed as SCLC by expert pathologists using hematoxylin and eosin (H&E) staining, based on the WHO classification 23.

Due to inadequate amounts of viable cancer cells, 20 cases were excluded from the study leaving 35 cases. Basis on TNM classification of malignant tumors 7th edition, all cases were staged. Specimens were snap-frozen in liquid nitrogen typically within 15 min after removal and stored at −80°C. Written informed consent for research was obtained from all patients, and our institutional review board approved the study plan. We collected clinicopathological details including neoadjuvant and adjuvant chemotherapy (NAC and AC, respectively), and listed them in Table 1.

**Table 1:** Comparisons of clinicopathological factors of all SCLC patients enrolled (*n* = 35) and those with high- and low expression of *HOTAIR*

	All cases, examined (*n* = 35)		Cases with high-*HOTAIR* expression (*n* = 12)		Cases with low-*HOTAIR* expression (*n* = 23)		
				
Factors	*N*	%	*N*	%	*N*	%	*P* value
Age (mean ± SD)	65.8 ± 6.60		63.3 ± 6.70		67.1 ± 6.30		0.10
Gender							
M	25	71.4	9	75.0	16	69.6	0.53
F	10	28.6	3	25.0	7	30.4	
Cumulative smoking (pack-years)	52 ± 31		52 ± 19		52 ± 36		0.96
Chemotherapy type							
NAC − AC−	3	8.60	1	8.33	2	8.70	
NAC + AC−	3	8.60	0	0	3	13.0	
NAC − AC+	23	65.7	8	66.7	15	65.2	
NAC + AC+	6	17.1	3	25.0	3	13.0	
Regimen							
NAC(*n* = 9)	CDDP + VP16 (8)	22.9	3	25.0	5	21.7	
	CDDP + DOC (1)	2.90	0	0	1	4.35	
AC(*n* = 29)	CDDP + VP16^1^ (22)	62.9	10	83.3	12	52.2	
	CBDCA + VP16^2^ (4)	11.4	0	0	4	17.4	
	Mix; 1 and 2 (3)	8.60	1	8.33	2	8.70	
Histological type (SCLC)							
Pure	24	68.6	11	91.7	13	56.5	0.04*
Combined	11	31.4	1	8.33	10	43.5	
	With Ad (5)	45.5	0	0	5	50.0	
	With LCC (2)	18.2	0	0	2	20.0	
	With LCNEC (4*)	36.4	1*	100	2	20.0	
	With others (1)	9.10	0	0	1	10.0	
	*include combined case (with both LCNEC/Ad)		*include combined case (with both LCNEC/Ad)				
Operation (SCLC)							
Partial resection	3	8.60	1	8.33	2	8.70	
Segmentectomy	0	0	0	0	0	0	
Lobectomy (1 lobe)	27	77.1	10	83.3	17	73.9	
Lobectomy + partial resection	1	2.90	1	8.33	0	0	
Lobectomy (2 lobes)	2	5.70	0	0	2	8.70	
Pneumectomy	2	5.70	0	0	2	8.70	
Pathological stage							
1a	11	31.4	1	8.33	10	43.5	
1b	4	11.4	2	16.7	2	8.70	
2a	7	20.0	3	25.0	4	17.4	
2b	2	5.70	0	0	2	8.70	
3a	10	28.6	6	50.0	4	17.4	
3b	1	2.90	0	0	1	4.35	
4	0	0	0	0	0	0	
Invasion (microscopic)							
Vascular invasion	29	82.9	9	75.0	20	87.0	0.33
Lymphatic invasion	20	57.1	10	83.3	10	43.5	0.03*
Relapse	15	42.9	8	66.7	7	30.4	0.04*
	Brain (6)	40.0	4	50.0	2	28.6	
	Lung (2)	13.3	2	25.0	0	0	
	Mediastinal LNs (4)	26.7	3	37.5	1	14.3	
	Stomach (1)	6.70	0	0	1	14.3	
	Liver (3)	20.0	1	12.5	2	28.6	
	Adrenal gl. (2)	5.70	1	12.5	1	14.3	
	Others (pleural eff.) (1)	6.70	0	0	1	14.3	
Survival							
Alive	19	54.3	5	41.7	14	60.9	
Dead	16	45.7	7	58.3	9	39.1	
	Lung cancer (11)	68.7	6	85.7	5	55.6	
	Other malignancy (2)	12.5	1	14.3	1	11.1	
	Other disease (2)	12.5	0	0	2	22.2	
	Unknown (1)	6.30	0	0	1	11.1	
DSS (mean ± SD)	45.3 ± 35.7 mos.		38.1 ± 27.2 mos		49.0 ± 39.5 mos		
RFS/DFS (SCLC) (mean ± SD)	40.9 ± 38.5 mos.		30.8 ± 30.7 mos		46.3 ± 41.6 mos		

SCLC, small-cell lung cancer; smoking index, a product of number of cigarettes per day by duration in years; NAC, neoadjuvant chemotherapy; AC, adjuvant chemotherapy; CDDP, cisplatin; CBDCA, carboplatin; VP-16, etoposide; DOC, docetaxel; Ad, adenocarcinoma; LCC, large cell carcinoma; LCNEC, large cell neuroendocrine carcinoma; LNs, lymph nodes; DSS, disease-specific survival; RFS, relapse-free survival; DFS, disease-free survival.

* *P* < 0.05.

Ten SCLC cell lines (COLO-668, COR-L51, COR-L88, DMS-79, DMS-53, Lu-134A, MS-1, SBC-3, SBC-5, and SBC-1), one adenocarcinoma cell line (A549) and a normal lung cell line (MRC-5), derived from embryonic normal lung tissue, were used. The former four lines were obtained from the European Collection of Cell Cultures, and the other were from Japanese Collection of Research Bio-resources or the RIKEN Bio Resource Center. All cells were maintained in the Dulbecco's modified Eagle's medium (DMEM) supplemented with 2 mmol/L l-alanyl-l-glutamine solution, 1.1% antibiotic-antimycotic mixed stock solution (Nacalai Tesque, Kyoto, Japan) and 10% fetal bovine serum (FBS), at 37°C, 5% CO_2_ incubator.

Primary-cultured normal bronchial epithelial (NBE) cells were obtained from fresh surgical materials. Emergence and proliferation of bronchial epithelial cells around the samples without any proliferation of fibroblasts and contamination was confirmed by microscopy.

### RNA preparation, reverse transcription, and quantitative real-time polymerase chain reaction

Total RNA from tissues and cells were extracted using the RNeasy mini kit or RNeasy mini kit plus (Qiagen, Tokyo, Japan). cDNAs were generated from 30 ng of total RNA. The resulting cDNA was subjected to a 45-cycle polymerase chain reaction (PCR) amplification step followed by quantitative real-time PCR (qRT-PCR) using the LightCycler 480 SYBR Green I Master protocol (Roche Applied Science, Indianapolis, IN). Triplicates were run for each gene for each sample as previously 16. Based on previous studies 24, the amount of *HOTAIR* RNA was normalized to that of beta-actin (*ACTB*) in tissue samples and xenografts, and to Glyceraldehydes-3-phosphate dehydrogenase (*GAPDH*) in cell lines and normal cells. The *HOTAIR/ACTB* ratio in 35 SCLC and 15 noncancerous lung tissues randomly chosen from the 35 patients were analyzed by qRT-PCR. Tumors were divided into two groups with high- and low expression based on *HOTAIR*/*ACTB* ratios, using receiver operating characteristic (ROC) curve analysis. The primer sequences are listed in Table S1.

### *HOTAIR* expression of SCLC cell lines as well as control cells

We assessed *HOTAIR* expression in above cell lines and normal controls, normalizing to *GAPDH*. To define high- and low-expression groups, we used the level of normal controls, that is, the cut-off level of high expression was defined as above those of normal controls.

### SCLC cell xenografts

We examined *HOTAIR* expression in xenografts as well 25. Four-week-old male nude mice *Crlj:CD1-Foxn1*^NU^ with ICR background were purchased from Charles River Laboratories, Japan, housed at the animal care facility of our institute and kept under standard temperature, humidity, and timed-lighting conditions and provided mouse chow and water ad libitum. The five SCLC cell lines with high expression (final concentration, 1.0 × 10^7^ cells/0.3 mL phosphate buffered saline [PBS] each) were injected directly into three sites of the abdominal subcutaneous adipose tissue of the mice in 0.3 mL of sterile PBS. Developed tumors were immediately frozen in liquid nitrogen and stored at −80°C until use.

### RNA interference

Cells were transfected with 20 nmol/L small interfering (si)RNAs targeting *HOTAIR*, using Lipofectamine RNAiMAX (Invitrogen, Carlsbad, CA) as per manufacturer's directions. Transfection efficiency was assessed using a fluorescence microscope (Leica-DMIRE2) following 72 h incubation after transfection of labeled positive control; BLOCK-iT Alexa Fluor Red Fluorescent Oligo (Invitrogen) according to manufacturer's procedures. Twenty nmol/L (final concentration) of siRNAs and 1.5 *μ*L of Lipofectamine RNAiMAX in a total volume of 101.5 *μ*L were used for transfection of SBC-3 cells in 24-well based analysis (transduction efficiency: 100%, knockdown efficiency: 50%). We transfected #1–3 si*HOTAIR* as previously 8,9,14 to SBC-3 cells. After 72 h, total RNAs were collected for qRT-PCR analysis. Primer sequences are listed in Table S1.

### Cell proliferation assay and matrigel invasion assay

For cell proliferation assays, 4.0 × 10^4^ cells were plated in triplicate on 24-well plates containing DMEM medium with 10% FBS, 1% antibiotics, and glutamine solution. Subsequently, the cell number was calculated using the Trypan-blue staining and an automated cell counter (TC10 Bio-Rad Laboratories, Tokyo, Japan) after transfection with siRNA for 24, 48, 72, and 96 h.

A matrigel invasion assay was performed using the Biocoat Matrigel Invasion Chamber (BD) according to the manufacturer's protocols. In brief, 4.0 × 10^4^ cells were plated in the upper insert chamber in serum-free medium. The bottom chamber contained DMEM medium with 10% FBS as an inducer of invasion. After 48 h, the bottom of the insert chamber was fixed and stained with Diff-Quick staining (Sysmex, Kobe, Japan). Cells on the dissected stained membrane were counted under a microscope. Each membrane was divided into four quadrants and the sum of all four quadrants was calculated. Each matrigel invasion assay was performed in triplicate, and compared with migration assays using control insert membranes.

### Comprehensive gene expression analysis using microarrays

Total RNAs from #1si*HOTAIR*-transfected SBC-3 cells and control cells (si*GFP*-transfected cells), were extracted using RNeasy mini kit Plus (Qiagen) and hybridized to the microarrays, Sure Print G3 Human GE 8 × 60K microarrays (Agilent Technologies, Santa Clara, CA), according to the manufacturer's instructions. Subsequently, data analysis was carried out using the GeneSpring GX 12 software (Agilent Technologies), with a stringency of *P* < 0.1 and a twofold or more change using gene ontology analysis.

### Statistical analysis

Continuous datasets were compared using an independent *t*-test between two groups, and categorical datasets were analyzed by the chi-square test. Significance of difference between two or more groups was estimated with the Mann–Whitney *U-*test or the Kruskal–Wallis test, as appropriate. Disease-specific survival (DSS) was defined by death only from SCLC. Relapse-free survival (RFS) was defined by metastasis as the first recurrence event. In this study, because second primary cancers were not found in all subjects, RFS is de facto disease-free survival (DFS). DSS and RFS/DFS curves were plotted according to the Kaplan–Meier method with the Cox–Mantel log-rank test applied for comparison. Univariate and multivariate analyses by the Cox proportional hazard method were performed. All differences were considered statistically significant at the level of *P* < 0.05, and there being a tendency at a level of *P* < 0.10. SPSS 19.0 (IBM Corporation, Somers, NY) was used for statistical analyses.

## Results

### Clinicopathological profiles of 35 SCLC cases

We assessed 35 surgically removed SCLC tumors and 15 lung tissues. The patients were mostly male, the average age was 65.8 years, and cumulative smoking was over 50 pack-years. Nine cases undertook chemotherapy before surgery, typically four courses of platinum (cisplatin [CDDP] or carboplatin [CBDCA]) plus etoposide (VP-16), and 26 (74%) cases did not have chemotherapy. Histologically, 69% (*n* = 24) were pure SCLC and vascular invasions were often observed (83%, *n* = 29). As expected, there were many stage I cases (*n* = 15, 43%). Generally, although SCLC patients once improved after chemotherapy, relapses frequently occur, resulting in poor prognosis. During the period of observation, 43% (*n* = 15) had relapses and 46% (*n* = 16) died. DSS and RFS/DFS were 45.3 ± 35.7 months and 40.9 ± 38.5 months, respectively.

### *HOTAIR* expressions and clinicopathological factors in SCLC

**Figure 1:**
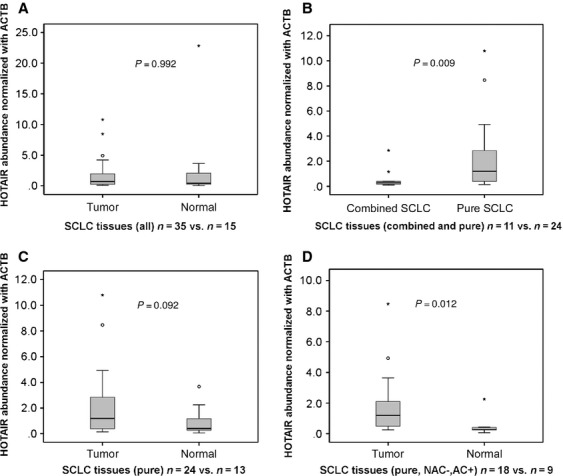
*HOTAIR* expression levels assessed by quantitative RT-PCR in 35 SCLC and normal tissues. Comparisons between all the tumors (*n* = 35) and normal tissues (*n* = 15) (A), pure (*n* = 24) and combined (*n* = 11) SCLC (B) (*P* = 0.009), pure SCLC and normal (C), and between pure SCLC with adjuvant chemotherapy and no neoadjuvant chemotherapy (*n* = 18) and normal (D) (*P* = 0.012) were made. *HOTAIR* was highly expressed in pure SCLC than combined SCLC and normal tissues.

We assessed *HOTAIR* expression levels in 35 tumors and 15 normal tissues by qRT-PCR normalized to the *ACTB*. *HOTAIR* levels between tumor tissues (*n* = 35) and normal tissues (*n* = 15) were not different significantly when analyzed across all cases (*P* = 0.992 [Mann-Whitney U test]; Fig. 1A). When compared between pure-SCLC tumor tissues (*n* = 24) and combined-SCLC tumor tissues (*n* = 11), *HOTAIR* expression was significantly higher in pure SCLC (*P* = 0.009; Fig. 1B). For pure-SCLC cases, *HOTAIR* had a tendency to be expressed more in tumor tissues (*n* = 24) than normal tissues (*n* = 13) (*P* = 0.092; Fig. 1C). Since chemotherapy prior to surgery might have some effect on cellular nature, analysis was limited to cases without such treatment (pure-NAC(−) cases), resulting in significantly higher expression in tumor (*n* = 18) than normal tissues (*n* = 9) (*P* = 0.012; Fig. 1D).

**Figure 2:**
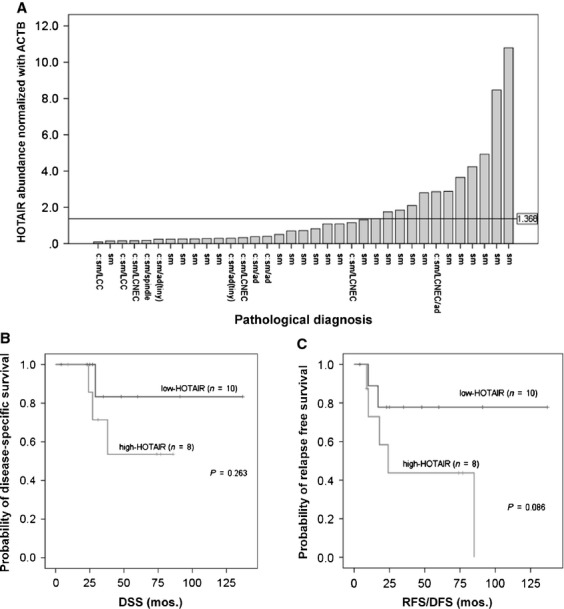
*HOTAIR* expression levels in SCLC and survival curves by *HOTAIR* expression. (A) *HOTAIR* expression in 35 SCLC and a reference level (*HOTAIR*/*ACTB* ratio = 1.368). (B and C) The Kaplan–Meier DSS curves and RFS/DFS curves, respectively, according to *HOTAIR* levels in pure-SCLC cases with adjuvant chemotherapy and no neoadjuvant chemotherapy (*n* = 18). Note that there was a tendency to poor prognosis in the high-expression group for the RFS/DFS of these groups (*P* = 0.086). For abbreviations, see text.

We divided 35 subjects by *HOTAIR* expression into two groups: high expression (*n* = 12) and low expression (*n* = 23), according to a *HOTAIR/ACTB* ratio of 1.368 in tumor tissues, obtained by the ROC method (Fig. 2A). The high-expression group contained significantly more pure SCLC (*P* = 0.04), more lymphatic invasion (*P* = 0.03), and more relapse (*P* = 0.04) than the low-expression group (Table 1). To perform survival analysis, we focused only on cases with pure SCLC, without NAC and with AC (*n* = 8 in high-expression group and *n* = 10 in low-expression group). The high-expression group tended to have lower survival for RFS/DFS (*P* = 0.086), but not for DSS (*P* = 0.263) (Fig. 2B and C). Univariate analyses of DSS in all the subjects revealed that there were tendencies toward poor prognosis in the cases with relapse and pathological stages II or higher (*P* = 0.058, 0.099, respectively, Table S2). Multivariate analyses of DSS demonstrated that AC (*P* = 0.005) and pathological stages (*P* = 0.02) were significant prognostic factors (Table S2). However, *HOTAIR* expression was not a prognostic factor by either uni- or multivariate analyses. In regard to RFS/DFS, multivariate analyses showed that AC and pathological stages were significant prognostic factors (*P* = 0.004 and 0.021, respectively) and there was a tendency to poor prognosis in the cases with high-*HOTAIR* expression (*P* = 0.071) as indicated in Table S2.

### Expression of *HOTAIR* in SCLC cells and their xenografts

**Figure 3:**
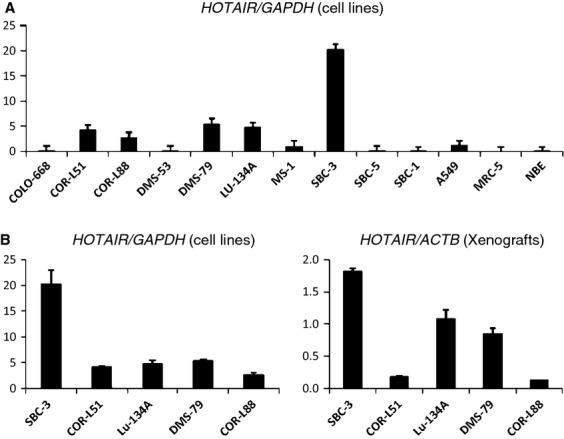
Expression of *HOTAIR* in cell lines and xenografts of SCLC. (A) Relative expression levels normalized to *GAPDH*. The SBC-3 line showed particularly high expression. (B) Comparisons in five SCLC cell lines with higher expression than normal cells. For abbreviations, see text.

We next examined *HOTAIR* expression in cell lines and normal cells. Normal cells had no expression. There were five SCLC cell lines (COR-L51, COR-L88, DMS-79, Lu-134A, and SBC-3) whose expression was significantly higher than normal, whereas the other five cell lines and the adenocarcinoma line showed the same low-level expression as normal cells. The SBC-3 cells expressed particularly highly (Fig. 3A). We made xenografts from these five cell lines and assessed *HOTAIR* expression using RT-PCR as normalized to *ACTB*. Although Lu-134A and DMS-79 lines expressed significantly highly compared with states of culture, it was SBC-3 that expressed most highly (Fig. 3B).

Consequently, we selected SBC-3 cells for further analyses. The SBC-3 line is an adherent cell line, others are not, and therefore RNAi was successful.

### Depletion of *HOTAIR* by siRNA leading to decreased proliferation and invasiveness

#1–3 siRNAs against *HOTAIR* gene and si*GFP* were conformed as described 8,9,14. All three siRNAs worked well and the gene expression was reduced significantly (Fig. 4A). In the proliferation experiments, however, depletion of *HOTAIR* by #1 si*HOTAIR* in SBC-3 cells dramatically decreased its proliferation ability whereas transfection of #2 or #3 si*HOTAIR* did not (Fig. 4B). Also, in the invasion assay, #1 si*HOTAIR* successfully reduced matrix invasiveness as compared with controls (si*GFP* transfection), whereas #2–3 siRNA did not (Fig. 4C).

**Figure 4:**
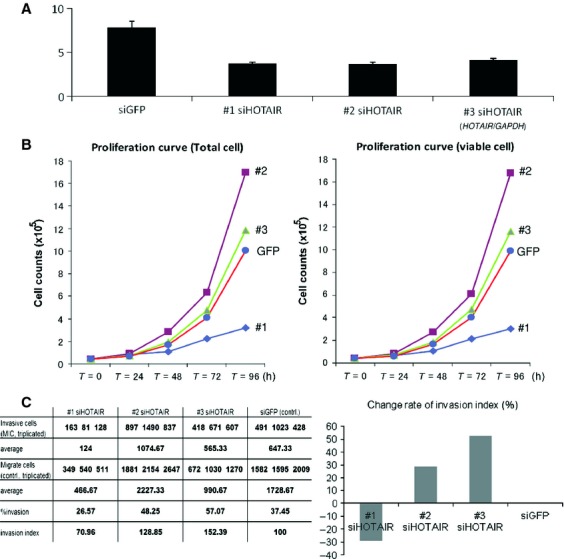
*HOTAIR* abundance, cellular proliferation and invasiveness in SBC-3 cells with transfection of siRNA and of siGFP. (A) The graph showed the *HOTAIR*/*GAPDH* ratio. #1–3 si*HOTAIR*s successfully reduced the expression levels. (B), #1 si*HOTAIR*-transfected cells reduced their ability of proliferation after 72 h, although #2, #3 si*HORAIR* did not reduce. (C) Invasiveness was assessed by % invasion and invasion index according to manufactures' protocol. In #1 si*HOTAIR*-transfected cells, almost 30% reduction of matrigel invasiveness was seen, whereas #2 and #3 si*HOTAIR* did not reduce invasiveness.

### Gene ontology analysis in si*HOTAIR*-transfected cells

We tested whether *HOTAIR* depletion by siRNA affected the pattern of gene transcriptions, especially in genes related to proliferation and invasiveness targeted by si*HOTAIR*. We succeeded in the transfection experiments using the SBC-3 cell line and subsequently performed gene expression analysis. Raw data of two samples (#1 si*HOTAIR*-transfected cells and si*GFP*-transfected cells) were deposited in the GEO database (GSE43877). We identified significantly altered 110 genes. Ontology analyses demonstrated that many of these genes were related to cell adhesion, proliferation, and mucin formation.

Among the upregulated genes, there were those related to cell adhesion such as *ASTN1* (*astrotactin 1*), *PCDHA1* and *10* (*protocadherin-alpha* [*Pcdha*] 1 and 10), and *CLDN11* (*Claudin-11*), and genes involved in mucin production including *MUC5AC* and *MUC4*. In addition, *ECM2*, coding an extracellular matrix protein, and *NRP2*, coding a transmembrane protein interacting with vascular endothelial growth factor, were among the top 15 upregulated genes (Table S3). The top 15 downregulated genes include *NTM*, neurotrimin, encoding a protein that may promote neurite outgrowth and adhesion, *PTK2B*, *protein tyrosine kinase* 2 *beta* (also known as *Pyk2*), which functions in the activation of MAPK signaling pathways, and *CTNNA2*, catenin (*cadherin-associated protein*) *alpha* 2, expression of which is important for maintaining a subset of neurons. In addition, *SIRPG* (*signal regulatory protein gamma*), a member of the immunoglobulin superfamily, known to be involved in the negative regulation of receptor tyrosine kinase-coupled signaling processes, and *ITGB8* (*Integrin beta-8*), coding a member of the integrin beta chain family, are downregulated. Generally, integrin complexes mediate cell–cell and cell–extracellular matrix interactions and play a role in human airway epithelial proliferation.

## Discussion

Among lincRNAs, *HOTAIR* is one of the most remarkable because of its relevance to metastases in common cancers such as breast and colon cancers. Here, we first demonstrated that *HOTAIR* was expressed in pure SCLC and higher expression was significantly related to lymphatic invasion and relapse. Multivariate analyses demonstrated that *HOTAIR* expression correlated with RFS. In vitro experiments demonstrated that half of SCLC cell lines expressed *HOTAIR* at higher levels than normal cells and that, using SBC-3 cells, knockdown of *HOTAIR* decreased proliferative activity and cellular invasiveness with altered expression of cell adhesion-related genes.

Accumulating reports suggest that *HOTAIR* is related to poorer prognosis of tumors. Several studies have shown a relationship between high *HOTAIR* expression and the poorer prognosis of diverse cancers 14–22, but there are few studies that reveal the importance of *Hox*-related genes and/or *HOTAIR* in SCLC because of the scarcity of fresh tissue samples. In fact, almost all evidence obtained for alterations of expression in *Hox-*related genes in SCLC to date was based on cell lines and their xenografts 26–29. To our knowledge, this is the first report on *HOTAIR* expression in primary SCLC tissues. In particular, we have shown that *HOTAIR* expression is significantly higher in SCLC tumors than normal tissues, considering induction chemotherapy (Fig. 1D), and that high expression of *HOTAIR* is relevant for relapse of SCLC. This tumor type is special for lung cancer, but proved to share similar characteristics of *HOTAIR*-related cellular regulations with ordinary carcinomas including those of breast and colon. Furthermore, our knockdown experiments of *HOTAIR* demonstrated that its depletion in SBC-3 cells caused altered expression of genes involved in neural cell adhesion, proliferation, and mucin formation although the findings are based on one cell line.

It is interesting, although not surprising, that *HOTAIR* correlates with SCLC proliferation and invasion with expression of genes implicated in cell adhesion and mucin production. In fact, depletion of *HOTAIR* upregulates genes implicated with cellular adhesion and mucin production including *ASTN1*, *PCDHA1*, and *MUC5AC*. The roles of each of these genes have been elucidated to some extant: *ASTN1*, is required for appropriate and timely migration of cerebellar granule cells 30; *PCDHA1* belongs to the cadherin superfamily and mediate the formation and maintenance of specific synaptic connections 31; *MUC5AC*, whose expression of protein and mRNA is significantly decreased in gastric cancer tissue 32. On the other hand, the depletion of *HOTAIR* downregulates some genes implicated in tumor invasion and proliferation; *NTM* is expressed in fetal brain at higher levels than that in mature brain and is more highly expressed in nervous tumors than that in normal brain tissues 33; *PTK2B* mediates cell proliferation and invasiveness in HCC cells by upregulation of the *c-Src* and ERK/MAPK-signaling pathway, and is related to progression and metastasis in breast cancer, together with focal adhesion kinase 34,35. Genes with fold change values of 10 or more were including genes mainly relevant to neural development (*ASTN1*, *PCDHA1*, *MUC5AC*, and *NTM*). Our data suggest that *HOTAIR* mainly regulates the expression of genes related to neural development in SCLC cells.

Possible limitations of our analysis and interpretation are as follows: First, our analyses were based on surgically resectable cases representing a very minor population. In fact, several reports indicated that patients with surgically resectable SCLC had much better prognosis and showed 5-year survival rate 30–70% 36–38, and we were not able to show significantly different survival between the groups with low- and high-*HOTAIR* expression (Fig. 2B and C). However, if we only used material from inoperable cases, in other words, the majority of SCLC patients, almost all patients were of extended disease, and as a result, we had almost no patients with good prognosis. Certainly, we need more cases with good prognosis to obtain significant difference of prognosis between low- and high-*HOTAIR* groups. Second, we succeeded in RNAi experiments using only the SBC-3 cells. As mentioned above, the SBC-3 cell line was established from a bone metastasis and not from a primary site. It is known that *HOTAIR* expression in metastatic sites was higher than in primary sites for breast cancer 14. Possibly, high expression in the SBC-3 cells may be due to its metastatic nature, rather than its neuroendocrine nature. Further analysis using other cell lines is warranted although RNAi experiments will be a challenge.

Finally, although the knockdown efficacy was similar in #1–3 si*HOTAIRs*, suppression of cell proliferation and invasion were induced only by #1 si*HOTAIR*. This might be because the time to onset of gene silencing only by #1 siRNA was appropriate, and because the sequence conditions of the three siRNAs were different. In particular for the latter point, following four sequence conditions were suggested to give rise to highly effective RNAi in mammalian cells 39,40: (1) the 5′ antisense-strand (AS) end, A or U; (2) the 5′ sense-strand (SS) end, G or C; (3) the 5′-terminal one third of AS, A/U-rich; and (4) a long G/C stretch, absent from the 5′-terminal two thirds of SS. Considering these conditions as compared among #1–3 si*HOTAIRs*, only #1 si*HOTAIR* met the four conditions at the same time.

si*HOTAIR* may represent a new therapeutic target for SCLC, in particular, of its metastatic phase. Although several kinds of pharmaceutical agents are available for SCLC treatment, *HOTAIR* therapy may be useful after the development of multidrug resistance. However, we have to bear in mind that biological, pathological, and clinical evidence for SCLC on *HOTAIR* is very limited and that our knowledge of homeobox genes, the target genes of *HOTAIR*, is very sparse. Further analysis of *HOTAIR* expression in systemic tissues of both adults and fetuses followed by further cell line experiments and in vivo analyses on tissue effects by siRNA administration using a more number of siRNA will be required.

## Supporting Information

**Table S1.** Primer sequences of RT-PCR for *HOTAIR* quantification as well as *GAPDH* and *ACTB* as controls. Also, primers for RNAi experiments are shown. **Table S2.** Univariate and multivariate analysis of DSS and RFS/DFS. **Table S3.***HOTAIR* expression mainly associated with genes contributes to cell adhesion.
