# Supplementary material for: Long noncoding RNA HOTAIR is relevant to cellular proliferation, invasiveness, and clinical relapse in small-cell lung cancer
Source: Cancer Med. 2014 Mar 3;3(3):632–42. doi: 10.1002/cam4.220 (PMC4101754; doi:10.1002/cam4.220)
Supplement: Supplementary file 1 — Table S1. Primer sequences of RT-PCR for HOTAIR quantification as well as GAPDH and ACTB as controls. Also, primers for RNAi experiments are shown. Table S2. Univariate and multivariate analysis of DSS and RFS/DFS. Table S3.HOTAIR expression mainly associated with genes contributes to cell adhesion. [file cam40003-0632-SD1.docx]

**Table S1.** Primer sequences of RT-PCR for *HOTAIR* quantification as well as GAPDH and ACTB as controls. Also, primers for RNAi experiments are shown.

*HOTAIR* primers

*HOTAIR* (F): 5’-GGTAGAAAAAGCAACCACGAAGC-3’

*HOTAIR* (R): 5’-ACATAAACCTCTGTCTGTGAGTGCC-3’

*GAPDH* (F): 5’-CCGGGAAACTGTGGCGTGATGG-3’

*GAPDH* (R): 5’-AGGTGGAGGAGTGGGTGTCGCTGTT-3’

*ACTB* (F): 5’-AGAAAATCTGGCACCACACC-3’

*ACTB* (R): 5’-AGAGGCGTACAGGGATAGCA-3’

siRNAs targeting *HOTAIR* and *GFP*

#1 si*HOTAIR*,

sense: 5’-GAACGGGAGUACAGAGAGAUU-3’

antisense: 3’-UUCUUGCCCUCAUGUCUCUCU-5’

#2 si*HOTAIR*,

sense: 5’-CCACAUGAACGCCCAGAGAUU-3’

antisense: 3’-UUGGUGUACUUGCGGGUCUCU-5’

#3 si*HOTAIR*,

sense: 5’-UAACAAGACCAGAGAGCUGUU-3’

antisense: 3’-UUAUUGUUCUGGUCUCUCGAC-5’

si*GFP*

sense: 5’-CUACAACAGCCACAACGUCdTdT-3’

antisense: 3’-TTGAUGUUGUCGGUGUUGCAG-5’

**Table S2.** Univariate and multivariate analysis of DSS and RFS / DFS.

| Table S2. |  |  |  |  |  |  |  |  |
| --- | --- | --- | --- | --- | --- | --- | --- | --- |
| Univariate and multivariate analyses for disease-specific survival (DSS) and relapse-free survival / disease free survival (RFS / DFS). | | | | | | | | |
| A. Univariate analysis and multivariate analysis for DSS of all SCLC cases (n=35) | | | | |  |  |  |  |
| Factors |  | univariate analysis | | |  | multivariate analysis | | |
|  |  | Relative risk | 95%CI | P |  | Relative risk | 95%CI | *P* |
| Pathological type (combined/pure) |  | 1.51 | 0.440-5.16 | 0.514 |  | - |  | - |
| Age (≥65/<65) |  | 1.56 | 0.455-5.33 | 0.480 |  | - |  | - |
| Sex (Male/Female) |  | 0.96 | 0.254-3.62 | 0.952 |  | - |  | - |
| Cumulative smoking (pack-year) (≥50/<50) | | 1.7 | 0.451-6.43 | 0.432 |  | - |  | - |
| Neoadjuvant chemo (none/done) | | 0.67 | 0.196-2.29 | 0.523 |  | - |  | - |
| Adjuvant chemo (none/done) | | 2.99 | 0.787-11.4 | 0.108 |  | 18.9 | 2.49-144 | 0.005a |
| Recurrence or relapse (positive/negative) | | 202 | 0.839-4.87x10^4^ | 0.058a |  | - |  | - |
| Pathological Stage (pathological St. ≥II/<II) | | 3.65 | 0.784-17.0 | 0.099a |  | 11.9 | 1.48-95.0 | 0.02a |
| Vascular invasion (positive/negative) | | 2.8 | 0.357-21.9 | 0.327 |  | - |  | - |
| Lymphatic invasion (positive/negative) | | 0.93 | 0.284-3.06 | 0.908 |  | - |  | - |
| Metastasis (positive/negative) | | 1.96 | 0.571-6.71 | 0.286 |  | - |  | - |
| Emphysema (positive/negative) | | 1.11 | 0.337-3.65 | 0.865 |  | - |  | - |
| Interstitial pneumonia (positive/negative) | | 1.07 | 0.284-4.03 | 0.921 |  | - |  | - |
| HOTAIR/ACTB (≥1.368/<1.368) | | 1.58 | 0.481-5.18 | 0.451 |  | 2.05 | 0.543-7.73 | 0.29 |
|  |  |  |  |  |  |  |  |  |
| B. Univariate analysis and multivariate analysis for RFS/DFS of all SCLC cases (n=35) | | | | | |  |  |  |
| Factors |  | univariate analysis | | |  | multivariate analysis | | |
|  |  | Relative risk | 95%CI | P |  | Relative risk | 95%CI | *P* |
| Pathologcal type (combined/pure) |  | 1.2 | 0.410-3.53 | 0.738 |  | - |  | - |
| Age (≥65/<65) |  | 1.15 | 0.404-3.25 | 0.799 |  | - |  | - |
| Sex (Male/Female) |  | 1.1 | 0.348-3.48 | 0.869 |  | - |  | - |
| Cumulative smoking (pack-year) (≥50/<50) | | 1.2 | 0.410-3.52 | 0.738 |  | - |  | - |
| Neoadjuvant chemo (none/done) | | 0.94 | 0.300-2.97 | 0.922 |  | - |  | - |
| Adjuvant chemo (none/done) | | 2.37 | 0.751-7.49 | 0.141 |  | 13.6 | 2.29-80.1 | 0.004a |
| Pathological Stage (pathological St. ≥II/<II) | | 2.44 | 0.774-7.70 | 0.128 |  | 6.76 | 1.33-34.5 | 0.021a |
| Vascular invasion (positive/negative) | | 1.68 | 0.378-7.45 | 0.496 |  | - |  | - |
| Lymphatic invasion (positive/negative) | | 1.35 | 0.478-3.81 | 0.571 |  | - |  | - |
| Metastasis (positive/negative) | | 2.12 | 0.745-6.01 | 0.159 |  | - |  | - |
| Emphysema (positive/negative) | | 0.68 | 0.231-2.00 | 0.484 |  | - |  | - |
| Interstitial pneumonia (positive/negative) | | 1.91 | 0.677-5.36 | 0.222 |  | - |  | - |
| HOTAIR/ACTB (≥1.368/<1.368) | | 1.93 | 0.697-5.34 | 0.206 |  | 2.94 | 0.903-9.58 | 0.071a |
|  |  |  |  |  |  |  |  |  |
| C. Univariate analysis and multivariate analysis for DSS of pure SCLC cases (n=18) | | | | | |  |  |  |
| Factors |  | univariate analysis | | |  | multivariate analysis | | |
|  |  | Relative risk | 95%CI | P |  | Relative risk | 95%CI | *P* |
| Age (≥65/<65) |  | 1.13 | 0.118-10.9 | 0.913 |  | - |  | - |
| Sex (Male/Female) |  | 0.68 | 0.069-6.59 | 0.735 |  | - |  | - |
| Cumulative smoking (pack-year) (≥50/<50) | | 2.21 | 0.228-21.3 | 0.494 |  | - |  | - |
| Recurrence or relapse (positive/negative) | | 129 | 0.025-6.60x10^5^ | 0.264 |  | - |  | - |
| Pathological Stage (pathological St. ≥II/<II) | | 1.47 | 0.152-14.3 | 0.739 |  | 0.970 | 0.092-10.2 | 0.980 |
| Vascular invasion (positive/negative) | | 0.52 | 0.054-5.05 | 0.574 |  | - |  | - |
| Lymphatic invasion (positive/negative) | | 1.47 | 0.152-14.3 | 0.739 |  | - |  | - |
| Metastasis (positive/negative) | | 3.2 | 0.324-31.5 | 0.320 |  | - |  | - |
| Emphysema (positive/negative) | | 1.48 | 0.208-10.6 | 0.695 |  | - |  | - |
| Interstitial pneumonia (positive/negative) | | 2.45 | 0.343-17.5 | 0.372 |  | - |  | - |
| HOTAIR/ACTB (≥1.368/<1.368) | | 3.38 | 0.351-32.5 | 0.292 |  | 3.35 | 0.32-35.0 | 0.312 |
|  |  |  |  |  |  |  |  |  |
| D. Univariate analysis and multivariate analysis for RFS/DFS of pure SCLC cases (n=18) | | | | | |  |  |  |
| Factors |  | univariate analysis | | |  | multivariate analysis | | |
|  |  | Relative risk | 95%CI | P |  | Relative risk | 95%CI | *P* |
| Age (≥65/<65) |  | 0.34 | 0.066-1.80 | 0.206 |  | - |  | - |
| Sex (Male/Female) |  | 0.42 | 0.076-2.34 | 0.323 |  | - |  | - |
| Cumulative smoking (pack-year) (≥50/<50) | | 1.68 | 0.324-8.68 | 0.538 |  | - |  | - |
| Pathological Stage (pathological St. ≥II/<II) | | 1.28 | 0.248-6.62 | 0.767 |  | 1.22 | 0.219-6.75 | 0.823 |
| Vascular invasion (positive/negative) | | 0.33 | 0.054-1.96 | 0.221 |  | - |  | - |
| Lymphatic invasion (positive/negative) | | 3.14 | 0.377-26.2 | 0.290 |  | - |  | - |
| Metastasis (positive/negative) | | 4.83 | 0.563-41.4 | 0.151 |  | - |  | - |
| Emphysema (positive/negative) | | 0.61 | 0.118-3.16 | 0.557 |  | - |  | - |
| Interstitial pneumonia (positive/negative) | | 2.85 | 0.636-12.7 | 0.171 |  | - |  | - |
| HOTAIR/ACTB (≥1.368/<1.368) | | 3.77 | 0.728-19.5 | 0.114 |  | 3.75 | 0.721-19.5 | 0.120 |
|  |  |  |  |  |  |  |  | a: *P*<0.10 |

**Table S3.** *HOTAIR* expression mainly associated with genes contributes to cell

adhesion.

| Table S3A |  |  |
| --- | --- | --- |
| up regulated genes [#1siHOTAIR/siGFP, p=0.1, Fold change≥2] | |  |
| **GeneSymbol** | **GeneName** | **Fold change(abs)** |
| ASTN1 | astrotactin 1 | 22.5 |
| PCDHA1 | protocadherin alpha 1 | 18.8 |
| MUC5AC | mucin 5AC, oligomeric mucus/gel-forming | 12.0 |
| ECM2 | extracellular matrix protein 2, female organ and adipocyte specific | 10.9 |
| MUC4 | mucin 4, cell surface associated | 9.26 |
| PCDHA10 | protocadherin alpha 10 | 8.61 |
| CCR8 | chemokine (C-C motif) receptor 8 | 7.82 |
| NRP2 | neuropilin 2 | 5.28 |
| AMBP | alpha-1-microglobulin/bikunin precursor | 5.08 |
| SIGLEC1 | sialic acid binding Ig-like lectin 1, sialoadhesin | 4.87 |
| PCDHA6 | protocadherin alpha 6 | 4.81 |
| GATA1 | GATA binding protein 1 (globin transcription factor 1) | 4.73 |
| CLDN11 | claudin 11 | 4.35 |
| COL6A3 | collagen, type VI, alpha 3 | 4.19 |
| CLDN22 | claudin 22 | 3.78 |
| NLGN3 | neuroligin 3 | 3.76 |
| PXN | Paxillin | 3.74 |
| SIGLEC14 | sialic acid binding Ig-like lectin 14 | 3.41 |
| NCAM1 | neural cell adhesion molecule 1 | 3.35 |
| HSPG2 | heparan sulfate proteoglycan 2 | 3.24 |
| NRP1 | neuropilin 1 | 3.23 |
| CLDN19 | claudin 19 | 3.21 |
| SELE | selectin E | 3.18 |
| MYBPC1 | myosin binding protein C, slow type | 3.12 |
| AGT | angiotensinogen (serpin peptidase inhibitor, clade A, member 8) | 3.07 |
| PVRL1 | poliovirus receptor-related 1 (herpesvirus entry mediator C) | 2.98 |
| CLDN3 | claudin 3 | 2.85 |
| BCAM | basal cell adhesion molecule (Lutheran blood group) | 2.80 |
| KAL1 | Kallmann syndrome 1 sequence | 2.76 |
| PLXNC1 | plexin C1 | 2.66 |
| IHH | Indian hedgehog | 2.65 |
| CNTNAP2 | contactin associated protein-like 2 | 2.60 |
| ADAM9 | ADAM metallopeptidase domain 9 | 2.59 |
| HAPLN4 | hyaluronan and proteoglycan link protein 4 | 2.58 |
| PTPRS | protein tyrosine phosphatase, receptor type, S | 2.56 |
| FREM3 | FRAS1 related extracellular matrix 3 | 2.56 |
| SRPX2 | sushi-repeat containing protein, X-linked 2 | 2.55 |
| NID2 | nidogen 2 (osteonidogen) | 2.51 |
| PCDHGB7 | protocadherin gamma subfamily B, 7 | 2.51 |
| PCDHGA12 | protocadherin gamma subfamily A, 12 | 2.48 |
| PKP4 | plakophilin 4 | 2.44 |
| CDHR5 | cadherin-related family member 5 | 2.43 |
| HSPG2 | heparan sulfate proteoglycan 2 | 2.42 |
| JAM3 | junctional adhesion molecule 3 | 2.39 |
| EFS | embryonal Fyn-associated substrate | 2.38 |
| EMB | Embigin | 2.37 |
| COL20A1 | collagen, type XX, alpha 1 | 2.36 |
| NRXN2 | neurexin 2 | 2.32 |
| SRCIN1 | SRC kinase signaling inhibitor 1 | 2.32 |
| CHST10 | carbohydrate sulfotransferase 10 | 2.31 |
| DCBLD1 | discoidin, CUB and LCCL domain containing 1 | 2.31 |
| NRCAM | neuronal cell adhesion molecule | 2.26 |
| ENTPD1 | ectonucleoside triphosphate diphosphohydrolase 1 | 2.23 |
| NFASC | Neurofascin | 2.17 |
| NRP2 | neuropilin 2 | 2.15 |
| SEMA5A | sema domain, seven thrombospondin repeats (type 1 and type 1-like), transmembrane domain (TM) and short cytoplasmic domain, (semaphorin) 5A | 2.15 |
| HSPB11 | heat shock protein family B (small), member 11 | 2.15 |
| PCDHGC3 | protocadherin gamma subfamily C, 3 | 2.13 |
| JAM2 | junctional adhesion molecule 2 | 2.10 |
| CDH23 | cadherin-related 23 | 2.10 |
| TROAP | trophinin associated protein (tastin) | 2.06 |
| GPR56 | G protein-coupled receptor 56 | 2.06 |
| CD96 | CD96 molecule | 2.06 |
| SEMA4D | sema domain, immunoglobulin domain (Ig), transmembrane domain (TM) and short cytoplasmic domain, (semaphorin) 4D | 2.04 |
| ITGB3 | integrin, beta 3 (platelet glycoprotein IIIa, antigen CD61) | 2.03 |
| LAMC3 | laminin, gamma 3 | 2.03 |
| COL16A1 | collagen, type XVI, alpha 1 | 2.02 |
| AMICA1 | adhesion molecule, interacts with CXADR antigen 1 | 2.01 |

(abs), absolute value

| Table S3B |  |  |
| --- | --- | --- |
| down regulated genes [#1siHOTAIR/siGFP, p=0.1, Fold change≥2] | |  |
| **GeneSymbol** | **GeneName** | **Fold change(abs)** |
| NTM | Neurotrimin | 39.6 |
| PTK2B | PTK2B protein tyrosine kinase 2 beta | 7.30 |
| AMTN | Amelotin | 6.51 |
| COL14A1 | collagen, type XIV, alpha 1 | 4.82 |
| CTNNA2 | catenin (cadherin-associated protein), alpha 2 | 4.49 |
| DEFB118 | defensin, beta 118 | 4.07 |
| IGFBP7 | insulin-like growth factor binding protein 7 | 3.79 |
| PCDHGC4 | protocadherin gamma subfamily C, 4 | 3.78 |
| CTNNA3 | catenin (cadherin-associated protein), alpha 3 | 3.65 |
| SIRPG | signal-regulatory protein gamma | 3.64 |
| PCDHGB4 | protocadherin gamma subfamily B, 4 | 3.61 |
| CLDN10 | claudin 10 | 3.47 |
| CDH10 | cadherin 10, type 2 (T2-cadherin) | 3.32 |
| ARVCF | armadillo repeat gene deleted in velocardiofacial syndrome | 3.17 |
| ITGB8 | integrin, beta 8 | 3.10 |
| SDK2 | sidekick homolog 2 (chicken) | 3.10 |
| OPCML | opioid binding protein/cell adhesion molecule-like | 2.80 |
| CCL4 | chemokine (C-C motif) ligand 4 | 2.70 |
| SPACA4 | sperm acrosome associated 4 | 2.69 |
| FERMT3 | fermitin family member 3 | 2.66 |
| NELL2 | NEL-like 2 (chicken) | 2.52 |
| FN1 | fibronectin 1 | 2.51 |
| FAT4 | FAT tumor suppressor homolog 4 (Drosophila) | 2.50 |
| PKP1 | plakophilin 1 (ectodermal dysplasia/skin fragility syndrome) | 2.44 |
| KITLG | KIT ligand | 2.42 |
| FER | fer (fps/fes related) tyrosine kinase | 2.39 |
| PCDH11Y | protocadherin 11 Y-linked | 2.38 |
| PCDH11X | protocadherin 11 X-linked | 2.38 |
| CNTNAP3 | contactin associated protein-like 3 | 2.32 |
| FBLN5 | fibulin 5 | 2.17 |
| PDPN | Podoplanin | 2.16 |
| CD24 | CD24 molecule | 2.15 |
| COL9A1 | collagen, type IX, alpha 1 | 2.14 |
| DSC3 | desmocollin 3 | 2.13 |
| ITGB2 | integrin, beta 2 (complement component 3 receptor 3 and 4 subunit) | 2.11 |
| BTBD9 | BTB (POZ) domain containing 9 | 2.09 |
| CDHR4 | cadherin-related family member 4 | 2.09 |
| COL28A1 | collagen, type XXVIII, alpha 1 | 2.07 |
| THBS3 | thrombospondin 3 | 2.05 |
| PARVG | parvin, gamma | 2.05 |
| AMIGO2 | adhesion molecule with Ig-like domain 2 | 2.01 |
| ARHGAP5 | Rho GTPase activating protein 5 | 2.00 |

(abs), absolute value
